# Diagnostic value of the percentage of glottic opening score for classifying videolaryngoscopy in children: a prospective validation study

**DOI:** 10.1111/anae.70233

**Published:** 2026-05-18

**Authors:** Phillip B. Sasu, Thorsten Dohrmann, Vera Köhl, Anne Mahler, Josephine Tolkmitt, Catharina Grün, Viktor A. Wünsch, Linda Krause, Christian Zöllner, Martin Petzoldt

**Affiliations:** ^1^ Department of Anaesthesiology, Centre for Anaesthesiology and Intensive Care Medicine University Medical Centre Hamburg‐Eppendorf Hamburg Germany; ^2^ Institute of Medical Biometry and Epidemiology University Medical Centre Hamburg‐Eppendorf Hamburg Germany

**Keywords:** airway management, laryngoscopy, respiratory system, tracheal intubation, videolaryngoscopy

## Abstract

**Introduction:**

The percentage of glottic opening (POGO) score quantifies the laryngeal view obtained during laryngoscopy. This secondary analysis of the prospective observational PeDiAC study aimed to compare the POGO score with subjective glottic view ratings on a visual analogue scale (VAS) for classifying difficult videolaryngoscopic tracheal intubation.

**Methods:**

Videolaryngoscopy was used as the first‐line approach for tracheal intubation in children over a study period of 16 months. Immediately after tracheal intubation, the airway operators documented a VAS rating of 0–100, with higher values indicating better glottic views. Using videolaryngoscopy recordings of airway management, POGO scores were assigned in a post‐hoc analysis.

**Results:**

The study comprised 904 tracheal intubations in 809 children. Difficult videolaryngoscopic tracheal intubation occurred in 47 (5.2%) cases. The POGO score had poorer diagnostic performance than VAS rating for classifying difficult videolaryngoscopic tracheal intubation, with AUROCs of 0.52 95%CI (0.42–0.62) and 0.79 (95%CI 0.73–0.86), respectively, p < 0.001. Based on the AUROC, the POGO score had a poorer diagnostic performance than VAS rating for predicting multiple laryngoscopy attempts (0.46 (95%CI 0.40–0.51) vs. 0.66 (95%CI 0.61–0.71), p < 0.001); multiple tracheal intubation attempts (0.45 (95%CI 0.41–0.50) vs. 0.64 (95%CI 0.60–0.68), p < 0.001); time to tracheal intubation > 90 s (0.45 (95%CI 0.40–0.51) vs. 0.70 (95%CI 0.65–0.75), p < 0.001); airway‐related adverse events (0.51 (95%CI 0.41–0.61) vs. 0.65 (95%CI 0.57–0.74), p = 0.030); and severe hypoxaemia (0.47 (95%CI 0.35–0.59) vs. 0.63 (95%CI 0.52–0.73), p = 0.047). A POGO score < 50% had low positive predictive value of 0.11 (95%CI 0.04–0.22) and sensitivity of 0.13 (95%CI 0.05–0.27) for the occurrence of a difficult videolaryngoscopic intubation and the POGO score showed limited inter‐rater reliability (intraclass correlation coefficient 0.61 (95%CI 0.47–0.72)).

**Discussion:**

The POGO score has poor diagnostic performance for classifying difficult videolaryngoscopic tracheal intubation and low inter‐rater reliability in children.

## Plain Language Summary

**What we did:**

We studied how anaesthetists look at a child's airway when putting in a breathing tube using a video camera tool. We compared two ways of scoring how easy or hard the view was. One method was called the POGO score, and the other was a simple rating scale where doctors gave a score from 0 to 100. We looked at over 900 operations in children who had breathing tubes inserted and checked which method better showed when the procedure was difficult.

**Why did we do it:**

Putting in a breathing tube can sometimes be hard, especially in children. Doctors need a good way to tell when it might be difficult so they can act quickly and keep patients safe. We wanted to find out which scoring method works best for spotting problems.

**What we found:**

We found that the simple rating scale worked much better than the POGO score for spotting difficult cases. The POGO score often missed problems and was not very reliable between different people using it. This means the simple scoring method may be a better and more useful way for doctors to judge how easy or hard it is to place a breathing tube in children.

## Introduction

Difficult tracheal intubation is a major contributor to anaesthesia‐related adverse events in children [1, 2, 3, 4]. There is growing evidence that videolaryngoscopy improves glottic view, increases the first‐attempt tracheal intubation success rate and reduces adverse events [4, 5, 6, 7, 8]. Videolaryngoscopy has been recommended as the first‐choice tracheal intubation technique in neonates and infants in recent guidelines [9].

The percentage of glottic opening (POGO) score is a quantitative assessment of the glottic view observed on laryngoscopy. It was designed to describe the view obtained on direct laryngoscopy in adults [10]. The POGO score quantifies the proportion of the glottis that is visible between the interarytenoid notch and the anterior commissure, i.e. 100%: entire glottis visible from the anterior commissure of the vocal cords to the interarytenoid notch; 0%: glottic opening and interarytenoid notch not visible [10]. The metric scale and the presumed objectivity of the POGO score have been proposed as advantages [10].

The POGO score is now used by some airway operators to describe the view obtained on videolaryngoscopy. However, a prospective study reported very low accuracy and only moderate inter‐rater reliability of the POGO score when used for videolaryngoscopy in adults [11].

It is currently unknown which POGO scores offer the best prospect of tracheal intubation success. A POGO score of < 50% has been used to define a restricted view with videolaryngoscopy [12, 13] and some authors have speculated that this might be an appropriate threshold for the use of tracheal intubation adjuncts [14]. While the POGO score has been used in paediatric practice [5, 15, 16, 17, 18], its diagnostic performance for assessing glottic visualisation and classifying difficult tracheal intubation using videolaryngoscopy has not been evaluated.

Visual analogue scales (VAS) are established tools to assess subjective ratings and are considered reliable measurement instruments [19, 20, 21]. They have been used in clinical studies to obtain reproducible subjective ratings of the quality of glottic view during laryngoscopy [4, 22, 23, 24, 25, 26].

This secondary analysis of the PeDiAC study [4, 27] aimed to determine whether the POGO scores assigned by an independent rater provided a superior classification of difficult videolaryngoscopic tracheal intubation compared with glottic view ratings assigned by the airway operators using a VAS. The study also evaluated the inter‐rater reliability of the POGO score and the predictive performance of the POGO score and VAS rating for specific airway‐related outcomes.

## Methods

The prospective observational PeDiAC study was conducted within a university children's hospital that utilised videolaryngoscopy as the first‐line approach for tracheal intubation during the study period [4]. Guardians provided written informed consent and the study was approved by the Ethics Committee of the Medical Association of Hamburg. We included eligible consecutive children (age < 18 y) who were scheduled for elective procedures in the operating theatre requiring general anaesthesia with tracheal intubation. We did not study patients in whom tracheal intubation using a flexible bronchoscope or an awake technique was planned. Details regarding screening for eligibility, allocation, outcome definitions and study conduct are reported elsewhere [4, 27].

We used C‐MAC™ videolaryngoscopes with reusable blades (Karl Storz, Tuttlingen, Germany) as the first‐line device for all elective tracheal intubations in the operating theatre. Standard (Miller or Macintosh) or hyperangulated (D‐blade) videolaryngoscope blades were used by airway operators at their discretion [8, 28, 29]. Airway operators were encouraged to obtain the best possible view of the glottic inlet in all children in accordance with institutional standards [4, 12, 27]. External laryngeal manipulation was permissible.

Immediately following tracheal intubation, the airway operators assessed tracheal intubation‐related factors and answered the question “*how good were the glottic view conditions?*” on a VAS ranging from 0 to 100 (0 – very poor glottic view; 100 – very good glottic view) [4]. The anonymised videos of all tracheal intubations were stored. The POGO scores were assigned on post‐hoc video analysis by an independent rater (PS) after completion of the primary PeDiAC study [4]. The POGO score was assigned to the best glottic view obtained during videolaryngoscopy. The rater was blinded to the VAS ratings of the airway operators, medical outcomes and healthcare data. The rater was able to review the videos in real‐time or frame by frame on a high‐resolution screen and to replay the videos. Five additional independent raters (TD, VK, AM, JT, VW) performed similar individual analyses of 100 videolaryngoscopy videos that were selected at random using a sequence created by Microsoft Excel (Microsoft Corp., Redmont, WA, USA).

The primary outcome was a difficult videolaryngoscopic tracheal intubation, defined as a difficult airway alert issued by the airway operators following videolaryngoscopy [27, 30, 31, 32]. No explicit criteria were used for issuing a difficult airway alert. Secondary outcomes were assessed by an independent member of the investigator group who was in attendance during induction of anaesthesia and airway management, but who was not involved in patient care. Secondary outcomes included multiple laryngoscopy attempts (with each blade insertion being one attempt); multiple tracheal intubation attempts (with each tracheal tube insertion by mouth or nose being one attempt) [4, 33, 34]; prolonged time to tracheal intubation (defined as > 90 s from first insertion of the device to intubation of the trachea); incidence of severe hypoxaemia (defined as oxygen saturations ≤ 70% or hypoxaemia‐related bradycardia); and incidence of airway‐related adverse events (defined as laryngospasm; bronchospasm; airway, oral, soft tissue or dental trauma; laryngeal swelling or use of corticosteroids to reduce swelling risk due to laryngeal manipulation; oesophageal intubation; pulmonary aspiration; or severe hypoxaemia). The PeDiAC score items and Cormack and Lehane grades were also documented by the airway operators [4, 27].

We hypothesised that the POGO scores assigned by the independent rater would achieve a higher diagnostic performance, expressed as area under the receiver operator characteristic curve (AUROC), for classifying difficult videolaryngoscopic tracheal intubation in children compared with the VAS ratings assigned by the airway operators. The sample size of this analysis was pre‐determined by the 904 tracheal intubations performed within the PeDiAC study. Based on the approach of Obuchowski and McClish [35], this sample size was adequate to detect a difference of ≥ 0.10 between AUROC values of the POGO score and VAS rating, with a power of 80%, a primary outcome prevalence of 5.2% [4, 27] and a two‐sided significance level of 5%.

Metric variables were evaluated for normal distribution by visual inspection of histograms. To compare outcomes between children with or without difficult videolaryngoscopic tracheal intubation, we applied the Mann–Whitney U test, Pearson's χ^2^ test with Yate's correction or Fisher's exact test, whichever was appropriate. The AUROCs were compared using the non‐parametric approach by DeLong et al. [36]. We determined optimal thresholds by calculating the Youden indices of the POGO score and VAS rating for the prediction of difficult videolaryngoscopic tracheal intubation. We calculated sensitivity, specificity, positive and negative predictive values for the occurrence of a difficult videolaryngoscopic tracheal intubation. We used a scatter plot to illustrate the concordance between POGO scores and subjective glottic view ratings on VAS. We calculated the intraclass correlation coefficient using a two‐way‐mixed effects model with an absolute agreement definition, to assess the agreement between the POGO score ratings of the six independent raters.

A two‐sided p value < 0.05 was regarded as significant for the primary outcome. Secondary outcomes were analysed descriptively; p values for secondary analyses are provided as descriptive summary measures and were not adjusted for multiplicity. Statistical analyses were performed using SPSS (version 29, IBM Inc., Armonk, NY, USA) and R (version 4.3.0, R Foundation for Statistical Computing, Vienna, Austria).

## Results

From April 2021 to August 2022, 904 videolaryngoscopic tracheal intubations were performed in 809 children within the PeDiAC study [4]. Median (IQR [range]) patient age was 4 (2–9 [0–18]) y, weight was 17 (11–32 [2–117]) kg and ASA physical status was 2 (1–3 [1–4]). Further details regarding patient characteristics are reported elsewhere [4]. Difficult videolaryngoscopic tracheal intubation was reported in 47 (5.2%) cases, with at least one airway‐related adverse event in 42 (4.6%). Multiple laryngoscopy attempts were required in 151 (16.7%) cases and multiple tracheal intubation attempts were required in 274 (30.3%) [4]. The PeDiAC score had an AUROC of 0.97 (95%CI 0.96–0.99) and the Cormack and Lehane classification had an AUROC of 0.69 (95%CI 0.62–0.76) for classifying difficult videolaryngoscopic tracheal intubation, as previously reported [4, 27]. The POGO scores were assigned using videos from 885 tracheal intubations; 19 videos could not be included due to missing, invalid, interrupted or incomplete video storage. The VAS ratings were complete, without missing values.

Median (IQR [range]) VAS ratings of the quality of glottic view differed between easy and difficult videolaryngoscopic tracheal intubation (95 (85–100 [0–100]) vs. 80 (50–90 [0–100]), respectively; p < 0.001) [4], unlike the POGO scores (80 (65–90 [0–100]) vs. 75 (60–93 [0–100]), respectively; p = 0.625) (Table 1).

**Table 1:** Outcomes of 904 videolaryngoscopic tracheal intubations in 809 children. Values are median (IQR [range]) or number (proportion).

	Overall	Difficult airway alert not issued	Difficult airway alert issued	p value
	n = 904	n = 857	n = 47	
POGO score	80 (65–90 [0–100])^#^	80 (65–90 [0–100])^‡^	75 (60–93 [0–100])^†^	0.625
Quality of glottic view; VAS rating	95 (80–100 [0–100])	95 (85–100 [0–100])	80 (50–90 [0–100])	< 0.001
Multiple laryngoscopy attempts	151 (16.7%)	116 (13.5%)	35 (74.5%)	< 0.001
Multiple tracheal intubation attempts	274 (30.3%)	238 (27.8%)	36 (76.6%)	< 0.001
Time to tracheal intubation > 90 s	150 (16.6%)^±^	117 (13.7%)^+^	33 (73.3%)^†^	< 0.001
≥ 1 airway‐related adverse events*	42 (4.6%)	22 (2.6%)	20 (42.6%)	< 0.001
Severe hypoxaemia*	32 (3.5%)	20 (2.3%)	12 (25.5%)	< 0.001
PeDiAC score	0 (0–1 [0–6])	0 (0–0 [0–4])	3 (2–4 [1–6])	< 0.001

POGO, percentage of glottic opening; VAS, visual analogue scale; PeDiAC, Paediatric Videolaryngoscopic Intubation and Difficult Airway Classification.

* See Methods for definitions.

^+^ One missing value.

^†^ Two missing values.

^±^ Three missing values.

^‡^ 17 missing values.

^#^ 19 missing values.

**Figure 1:**
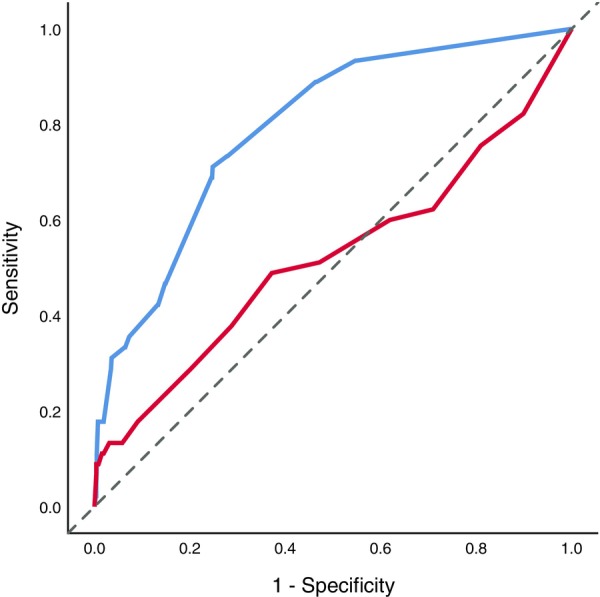
Receiver operating characteristic curves for the classification of difficult videolaryngoscopic intubation in 885 encounters. Red line, percentage of glottic opening (POGO) score; blue line, quality of glottic view rated on visual analogue scale (VAS).

The diagnostic performance of the POGO score was poorer than that of the subjective glottic view rating using VAS when classifying difficult videolaryngoscopic tracheal intubation (AUROC 0.52 (95%CI 0.42–0.62) vs. 0.79 (95%CI 0.73–0.86), p < 0.001) (Fig. 1). Optimal thresholds, determined by the best Youden index, were < 73% for the POGO score and < 84 for VAS rating (Table 2). At a POGO score threshold of < 50%, sensitivity was 0.13 (95%CI 0.05–0.27) and positive predictive value was 0.11 (95%CI 0.04–0.22) for the occurrence of a difficult videolaryngoscopic tracheal intubation. At the calculated optimal POGO score threshold of < 73%, sensitivity was 0.07 (95%CI 0.04–0.10) and positive predictive value was 0.49 (95%CI 0.34–0.64).

**Table 2:** Diagnostic accuracy of different thresholds of the percentage of glottic opening (POGO) score and subjective glottic view ratings on visual analogue scales (VAS) for the prediction of difficult videolaryngoscopic tracheal intubation. Values are number (95%CI).

		Sensitivity	Specificity	PPV	NPV	Youden index
**Threshold for POGO; %**	< 73*	0.49 (0.34–0.64)	0.63 (0.59–0.66)	0.07 (0.04–0.10)	0.96 (0.94–0.97)	0.12 (0.02–0.21)
	< 50	0.13 (0.05–0.27)	0.94 (0.92–0.96)	0.11 (0.04–0.22)	0.95 (0.94–0.97)	0.08 (0.00–0.15)
	< 25	0.09 (0.02–0.21)	0.99 (0.98–1.00)	0.36 (0.11–0.69)	0.95 (0.94–0.97)	0.08 (0.00–0.16)
**Threshold for VAS**	< 84*	0.71 (0.56–0.84)	0.75 (0.72–0.78)	0.13 (0.09–0.18)	0.98 (0.97–0.99)	0.46 (0.32–0.61)
	< 50	0.29 (0.16–0.44)	0.97 (0.95–0.98)	0.31 (0.18–0.47)	0.96 (0.95–0.97)	0.25 (0.13–0.38)
	< 25	0.11 (0.04–0.24)	1.00 (0.99–1.00)	0.56 (0.21–0.86)	0.95 (0.94–0.97)	0.11 (0.02–0.20)

PPV, positive predictive value; NPV, negative predictive value.

* Calculated optimal Youden index.

Based on the AUROCs POGO score had poorer diagnostic performance than VAS rating for predicting multiple laryngoscopy attempts, 0.46 (95%CI 0.40–0.51) vs. 0.66 (95%CI 0.61–0.71), p < 0.001; multiple tracheal intubation attempts, 0.45 (95%CI 0.41–0.50) vs. 0.64 (95%CI 0.60–0.68), p < 0.001; and time to tracheal intubation > 90 s, 0.45 (95%CI 0.40–0.51) vs. 0.70 (95%CI 0.65–0.75), p < 0.001; airway‐related adverse events (0.51 (95%CI 0.41–0.61) vs. 0.65 (95%CI 0.57–0.74), p = 0.030); and severe hypoxaemia (0.47 (95%CI 0.35–0.59) vs. 0.63 (95%CI 0.52–0.73), p = 0.047) (online Supporting Information Figure S1).

**Figure 2:**
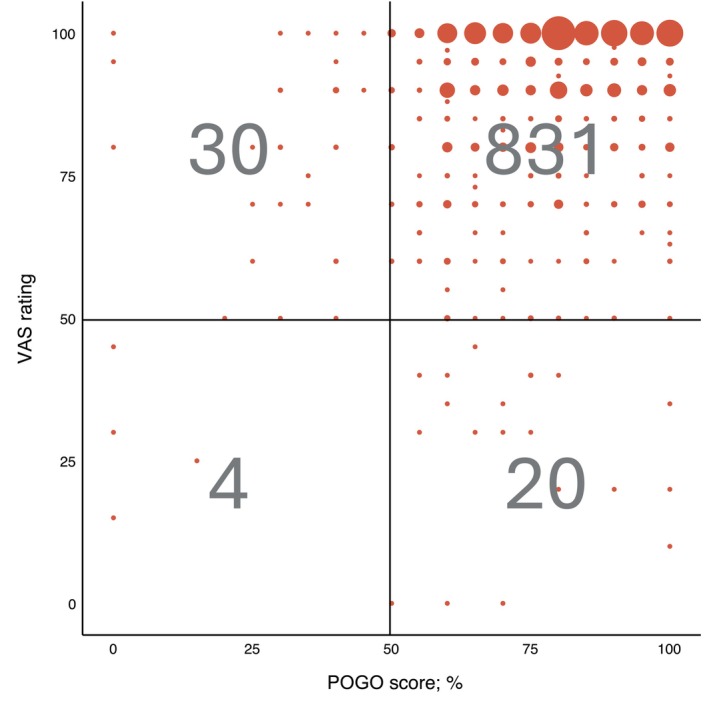
Concordance between the percentage of glottic opening score and visual analogue scale ratings. The size of the marker reflects the count of the respective values.

The concordance between POGO scores and VAS ratings was low, particularly at lower values (Fig. 2). A restricted glottic view, defined as a POGO score < 50% [10, 12, 14], was observed in 34/885 (3.8%) tracheal intubations, while a restricted glottic view, defined as a VAS rating < 50%, was observed in 25/885 (2.8%) tracheal intubations. Both definitions for restricted glottic view were fulfilled in four cases only (Fig. 2).

**Figure 3:**
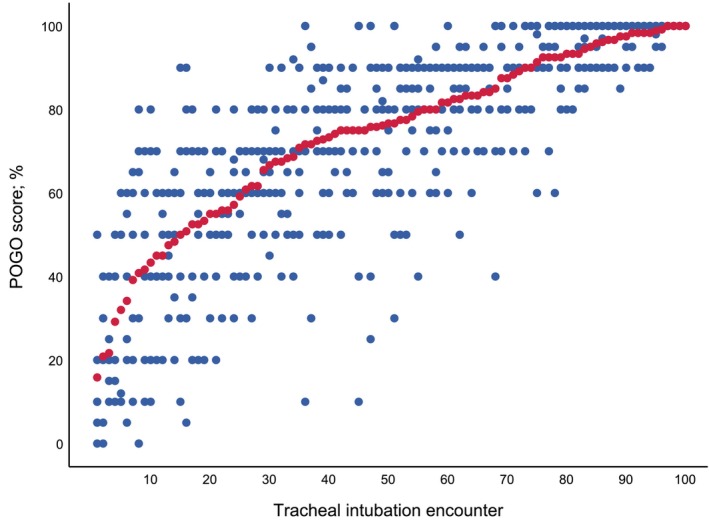
Distribution of the percentage of glottic opening (POGO) scores of six independent raters for 100 tracheal intubations using videolaryngoscopy, sorted by ascending mean POGO score values. Red, mean POGO score value of all six raters; blue, single POGO score ratings of the six raters.

We found only limited inter‐rater reliability for the POGO score between six independent raters, with an intraclass correlation coefficient of 0.61 (95%CI 0.47–0.72) [37]. This variability was particularly pronounced at lower POGO scores (Fig. 3).

## Discussion

In a large prospective paediatric cohort, a subjective assessment of the quality of glottic visualisation using VAS rating had greater diagnostic value than the POGO score when classifying difficult videolaryngoscopic tracheal intubation in children. The POGO score failed to discriminate between easy and difficult videolaryngoscopic tracheal intubations and showed no association with the number of laryngoscopy or tracheal intubation attempts, prolonged time to tracheal intubation or airway‐related adverse events.

The greatest discrimination between easy and difficult videolaryngoscopic tracheal intubations was found for a POGO score < 73%. As high POGO scores are often considered to reflect good tracheal intubation conditions [10, 12], this threshold may not be helpful for airway operators. However, it has also been suggested that tracheal intubation may be easier to perform if < 50% of the actual glottic opening is visualised. Two prospective studies in adults showed that deliberately restricted glottic views with POGO scores < 50% using hyperangulated videolaryngoscopy translated into faster and easier tracheal intubation [12, 13].

The limited inter‐rater reliability of the POGO score indicates that it is not an objective assessment tool for videolaryngoscopic tracheal intubation in children. There was particularly high variability in POGO score assignments at lower scores. Limited inter‐rater reliability of the POGO score has already been reported in adults [11].

Many factors might contribute to the low diagnostic accuracy and limited inter‐rater reliability of the POGO score. The score is assigned based on a visual approximation of distances between prespecified landmarks. Estimation by users also needs to consider the portion of this distance that cannot be seen [11]. This might explain the higher variance between raters at lower POGO score levels, because the ratio between the visible and non‐visible distance is particularly unfavourable.

In our study, the VAS rating perceived by the airway operator seldom reflected the corresponding POGO score determined by an independent rater. Our findings suggest that the quality of the glottic view on videolaryngoscopy is multifaceted and its classification requires a more comprehensive approach beyond estimating distances between anatomical landmarks [4, 27, 38, 39]. Although the POGO score and VAS rating are assigned on metric scales ranking from 0 to 100, there are important differences. While the POGO score is a quantitative assessment relying on approximations of distances between anatomical landmarks, the VAS rating does not rely on any objective criteria but solely on the subjective impressions of the airway operators regarding the quality of the glottic view. Therefore, VAS rating might comprise multiple facets of view restrictions, such as soiling, blood, secretions and oedema, that are not covered by the POGO score. The VAS rating also reflects the individual impression of the airway operator of whether a view restriction (e.g. POGO score 30%) might impact tracheal intubation meaningfully.

Previous difficult airway management is the strongest predictor of future difficulty [40, 41], reinforcing the importance of consistent documentation [27, 30, 32, 33]. The POGO score has not been validated for videolaryngoscopy in adults or children (i.e. determination of performance metrics in a representative cohort) [11, 42]. The VIDIAC score has been validated in children and outperformed the Cormack and Lehane classification when used to classify videolaryngoscopic tracheal intubation [27]; however, the diagnostic performance was less favourable than in adults [30, 32]. The PeDiAC score has been developed prospectively and validated for paediatric videolaryngoscopy and has shown high diagnostic performance [4, 27, 38]. Using unvalidated classifications or classifications with known poor diagnostic performance might result in inappropriate treatment decisions [33, 43, 44].

This study has some limitations. It took place in a single centre and equipment, protocols and patient populations differ between centres. All videolaryngoscopes used were produced by the same manufacturer. Rating the quality of glottic view on a VAS is not a validated assessment tool. The POGO scores were assigned under optimised, experimental conditions (post‐hoc video analysis by independent, blinded raters on a high‐resolution screen with permission to replay videos) and not by clinicians immediately after performing airway management. As the raters had access to the full video during the post‐hoc analysis, they could not be blinded to the passage of the tracheal tube through the laryngeal inlet. However, they were blinded to all further clinical data, assessments and medical outcomes of the respective patient, and none of the study outcome data were assessed in the video analysis. The absence of a difficult airway alert does not rule out minor incidences. The study did not evaluate the longitudinal prediction of the POGO score or VAS rating between a present and future tracheal intubation.

In conclusion, the POGO score had a poor diagnostic performance when used to classify difficult videolaryngoscopic tracheal intubation in children, showed no association with relevant airway‐related outcomes and had limited inter‐rater reliability.

## Supporting information


**Figure S1.** Receiver operating characteristic curves for the prediction of airway‐related outcomes in 885 tracheal intubations.
