# Supplementary material for: Diagnostic value of the percentage of glottic opening score for classifying videolaryngoscopy in children: a prospective validation study
Source: Anaesthesia. 2026 May 18;81(9):1228–35. doi: 10.1111/anae.70233 (PMC13446836; doi:10.1111/anae.70233)
Supplement: Supplementary file 1 [file ANAE-81-1228-s001.pdf]

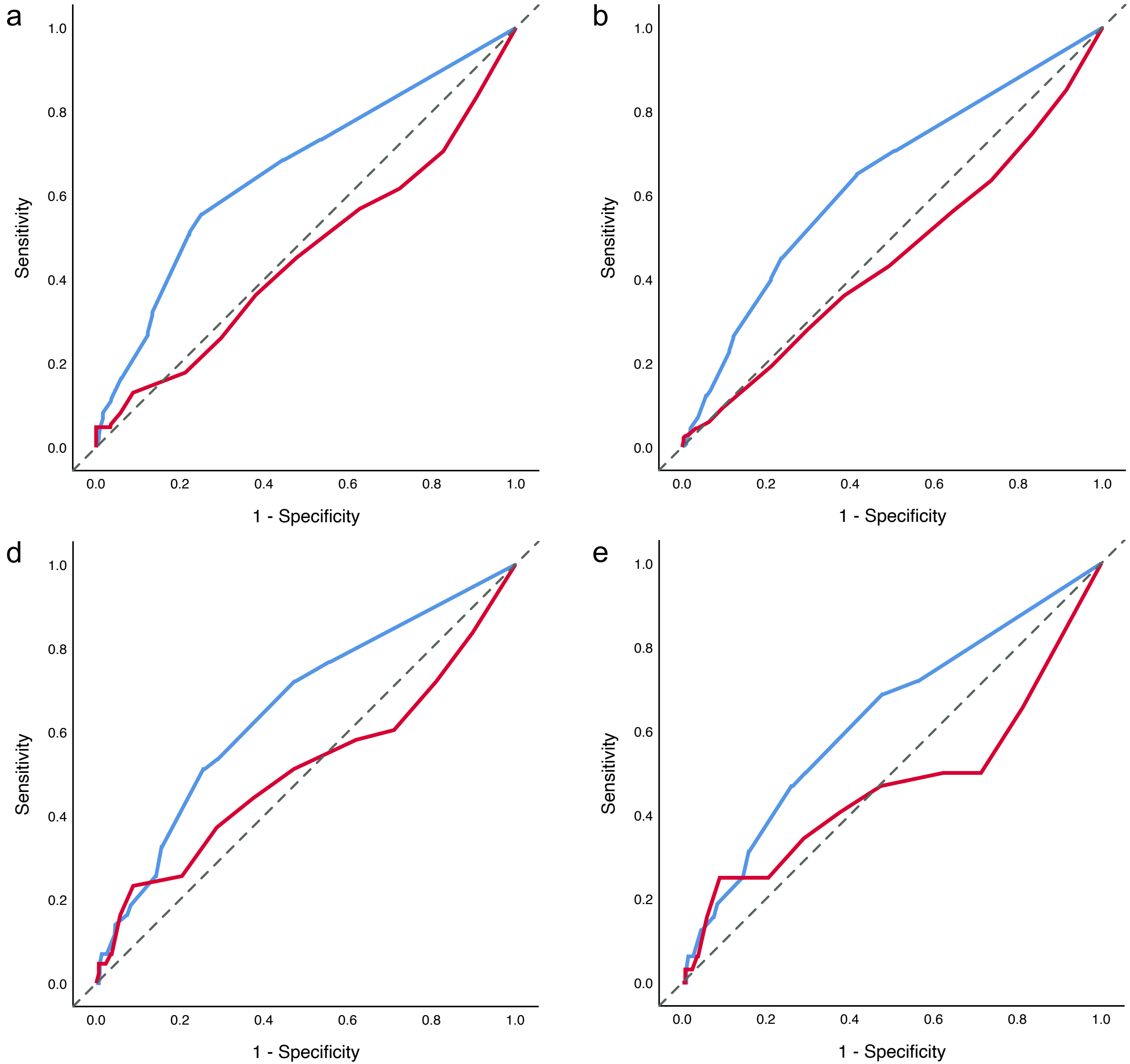

Figure S1 Receiver operating characteristic curves for the prediction of airway-related outcomes in 885 tracheal intubations: (a) multiple laryngoscopy attempts; (b) multiple intubation attempts; (c) time to tracheal intubation > 90 seconds; (d) airway-related adverse events; (e) severe hypoxaemia (SpO2 ≤ 70% or hypoxaemia-related bradycardia). Red line, percentage of glottic opening (POGO) score; blue line, quality of glottic view rated on visual analogue scales (VAS).
